# Development of a Novel Hierarchically Biofabricated Blood Vessel Mimic Decorated with Three Vascular Cell Populations for the Reconstruction of Small-Diameter Arteries

**DOI:** 10.1002/adfm.202300621

**Published:** 2023-11-03

**Authors:** Michele Carrabba, Marco Fagnano, Mohamed T Ghorbel, Filippo Rapetto, Bo Su, Carmelo De Maria, Giovanni Vozzi, Giovanni Biglino, Adam W. Perriman, Massimo Caputo, Paolo Madeddu

**Affiliations:** Bristol Heart Institute, School of Translational Health Sciences, Bristol Medical School, University of Bristol, Bristol BS2 8HW, UK; Bristol Heart Institute, School of Translational Health Sciences, Bristol Medical School, University of Bristol, Bristol BS2 8HW, UK; Cardiac Surgery, University Hospitals Bristol, NHS Foundation Trust Bristol BS2 8HW, UK; Bristol Dental School, University of Bristol, Bristol BS2 8HW, UK; Research Center “E. Piaggio”, University of Pisa Largo L. Lazzarino 1, 56122 Pisa, Italy; Department of Information Engineering, University of Pisa, Via G. Caruso 16, 56122 Pisa, Italy; Bristol Heart Institute, School of Translational Health Sciences, Bristol Medical School, University of Bristol, Bristol BS2 8HW, UK; Cardiorespiratory Unit, Great Ormond Street Hospital for Children, NHS Foundation Trust, London WC1N 3JH, UK; School of Cellular and Molecular Medicine, University of Bristol, Bristol BS8 1TD, UK; Bristol Heart Institute, School of Translational Health Sciences, Bristol Medical School, University of Bristol, Bristol BS2 8HW, UK; Cardiac Surgery, University Hospitals Bristol, NHS Foundation Trust Bristol BS2 8HW, UK; Bristol Heart Institute, School of Translational Health Sciences, Bristol Medical School, University of Bristol, Bristol BS2 8HW, UK

**Keywords:** 3D bioprinting, electrospinning, hierarchical biofabricated grafts, tissue engineering vascular grafts, vascular cells

## Abstract

The availability of grafts to replace small-diameter arteries remains an unmet clinical need. Here, the validated methodology is reported for a novel hybrid tissue-engineered vascular graft that aims to match the natural structure of small-size arteries. The blood vessel mimic (BVM) comprises an internal conduit of co-electrospun gelatin and polycaprolactone (PCL) nanofibers (corresponding to the tunica intima of an artery), reinforced by an additional layer of PCL aligned fibers (the internal elastic membrane). Endothelial cells are deposited onto the luminal surface using a rotative bioreactor. A bioprinting system extrudes two concentric cell-laden hydrogel layers containing respectively vascular smooth muscle cells and pericytes to create the tunica media and adventitia. The semi-automated cellularization process reduces the production and maturation time to 6 days. After the evaluation of mechanical properties, cellular viability, hemocompatibility, and suturability, the BVM is successfully implanted in the left pulmonary artery of swine. Here, the BVM showed good hemostatic properties, capability to withstand blood pressure, and patency at 5 weeks post-implantation. These promising data open a new avenue to developing an artery-like product for reconstructing small-diameter blood vessels.

## 1 Introduction

Cardiovascular disease (CVD), including coronary heart disease (CHD) and peripheral arterial disease (PAD), is the leading cause of death worldwide, with an estimated mortality incidence of 17.9 million people in 2019.^[1]^ In Europe, CVD is the primary cause of premature death for 2.2 million females and 1.9 million males aged <70.^[2]^ Moreover, patients with CHD or PAD suffer poor quality of life and require multidrug treatments, frequent hospitalizations, and revascularization. Coronary artery bypass grafting is the most common cardiac surgery procedure worldwide, with annual volumes of about 200000 cases in the US and an average incidence rate of 62 per 100 000 inhabitants in Western European countries.^[3]^

Autologous vessels currently remain the preferred solution for bypass surgery. The saphenous vein (SV) and internal thoracic artery represent the gold standard for grafting small-diameter blood vessels, such as coronary, infra-inguinal, and infra-geniculate arteries. However, invasive harvesting and limited availability remain significant burdens. Moreover, the patency rate remains suboptimal, with coronary reconstructions showing failure rates from 50 to 80% at 8–10 years follow-up.^[4,5]^ Synthetic vascular grafts, such as Dacron and Polytetrafluoroethylene (PTFE), showed satisfactory results for replacing large- and medium-diameter blood vessels, such as the carotid or common femoral artery.^[6]^ However, their failure rate was high when applied to small-diameter artery disease. Thrombotic events and restenosis make reintervention necessary in 50% of the cases by the end of the year.^[7–9]^ Decellularized native tissues are also available. However, decellularization can weaken the graft’s mechanical properties, predisposing it to aneurysmal dilatation. Vice versa, incomplete decellularization can trigger an immunogenic response by the graft recipient and rejection.^[10]^

Small-diameter, tissue-engineered vascular grafts (TEVG) fabricated with biodegradable materials, able to confer temporary support for cell growth, could solve the lack of autologous grafts.^[11–13]^ The ideal TEVG should reproduce the complex structure and anti-thrombotic, contractile, and deformation-resistant properties of a native artery.^[14,15]^ Since the early 2000s, various acellular and cellularized grafts have been proposed. Encouraging results were reported by L’Heureux, who applied an autologous cell-sheet-based graft in clinical trials. Nonetheless, the production time turned out to be ≈7.5 months.^[16]^ Biodegradable synthetic conduits seeded with endothelial cells (ECs) showed an improved patency rate but did not surpass autologous grafts.^[17,18]^ Hybrid TEVGs, combining synthetic and natural materials properties, can be fabricated by reinforcing decellularized native vessels with polycaprolactone (PCL).^[19]^ PCL-collagen grafts seeded with ECs, vascular smooth muscle cells (VSMCs), and fibroblasts proved promising. However, the long conditioning period was a significant constraint.^[20]^ Moreover, the outcome was suboptimal, with frequent intima hyperplasia, thrombotic events, and loss of patency.^[21–27]^ A multi-layered graft containing mesenchymal cells was fabricated through a rapid automated process, demonstrating good hemocompatibility and vascular implantability in two rabbits. However, mechanical properties were still inappropriate.^[28]^ In essence, there is no TEVG suitable for small-diameter artery substitution.

Here, we report the production and testing of a novel blood vessel mimic (BVM), hierarchically organized to match the natural structure of human arteries. The supporting material comprises an internal layer of co-electrospun gelatin (GL) and PCL nanofibers (corresponding to the tunica intima of an artery), surrounded by additional PCL-aligned nanofibers (the elastic membrane), respectively providing an adhesive luminal surface and mechanical strength to the structure. A precise topographic seeding of specialized vascular cells completes the BMV: ECs facing the lumen create an anti-thrombogenic surface, while VSMCs and pericytes (PCs) loaded in two concentric layers of bio-printed Sodium Alginate and Pluronic (AG/PL) gel populate the tunica media and adventitia. The whole process was optimized to minimize graft manipulation by the user and reduce production time. The complete BVM was finally assembled, cultured in dynamic conditions, and implanted in pigs after quality evaluation of the manufacturing procedure, using surgical procedures applied for vascular reconstruction in patients.

## 2 Results

### 2.1 Acellular Graft Production / Internal Polymeric Core Fabrication and Characterization

The polymeric conduit forming the core of the hybrid vascular graft was fabricated to fulfill two fundamental features, creating an internal biocompatible surface and being able to withstand blood pressure. To this aim, three conduits were fabricated and tested: the first made of GL nanofibers (named R-GL); the second of co-electrospun GL/PCL nanofibers and additional PCL fibers (R-GL/PCL); and the third of co-electrospun GL/PCL nanofibers and additionally aligned PCL fibers (C-GL/PCL).

**Figure 1:**
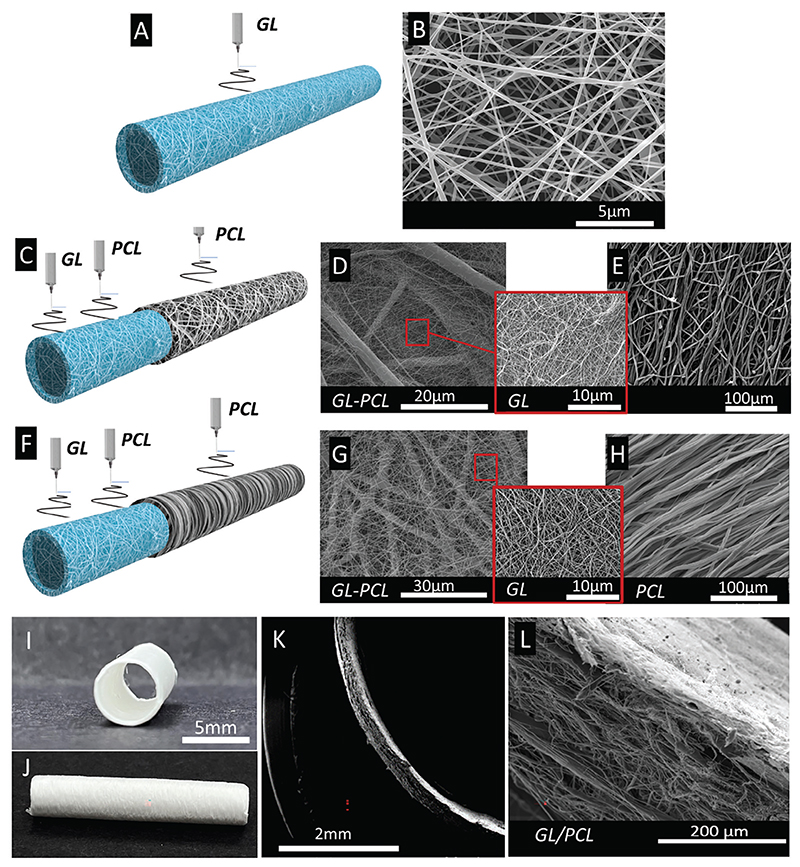
Fabrication of three polymeric conduit prototypes using electrospinning. A) Schematic render of the R-GL polymeric conduit. B) Representative SEM image of GL nanofibers produced for the R-GL. C) Schematic render of the R-GL/PCL conduit, showing an internal layer of co-electrospun GL and PCL and an external layer of randomly aligned PCL fibers. D,E) Representative SEM images of the internal layer of GL and PCL nanofibers D) and external PCL nanofibers E). F) Schematic render of the C-GL/PCL conduit, showing an internal layer of co-electrospun GL and PCL and an external layer of circumferentially aligned PCL fibers. G,H) Representative SEM images of the internal layer of GL and PCL nanofibers G) and externally aligned nanofibers of PCL H). I,J) Representative image of the C-GL/PCL tubular scaffold. K,L) SEM pictures of the scaffold cross-section K) and magnification of the wall section L).

The R-GL conduit was created by electrospinning GL nanofibers around a cylindrical rotating mandrel with an outer diameter of 5 mm and crosslinking them with glycidyloxy-propyltrimethoxy-silane (GPTMS) to improve resistance in aqueous conditions (Figure 1A). The highly biocompatible mesh of GL, possessing natural ECM binding proteins, confers an ideal surface for cell adhesion.^[29]^ Post-process analyses carried out with scanning electron microscopy (SEM) showed an overall thickness of 540±40 μm, GL fibers dimension of 90±18 nm, and pore size of 290±23 nm (Figure 1B).

For the R-GL/PCL conduit, PCL nanofibers were added to confer further mechanical resistance and durability (Figure 1C).^[30]^ An intima-like layer was created by electrospinning GL and PCL simultaneously onto a rotating cylindrical collector. Post-process analysis showed that GL and PCL fibers (90±18 nm and 0.87±0.12 μm in size, respectively) formed an isotropic nanofibrous surface with a porosity of 27±2% and pore size of 290±23 nm (Figure 1D). The values of this innermost layer are compatible with proper nutrient exchange and ECs adhesion.^[31–33]^ In addition, the co-electrospinning stage allowed to create an interconnected mesh of fibers, thereby reducing the risk of delamination. This first layer was then reinforced with a consecutive deposition of the PCL electrospun fibers mimicking the lamina elastica that separates the tunica intima from the media in an artery. This demarcating element also has the putative function of impeding cell invasion from outer layers into the intima. SEM analyses showed randomly aligned PCL fibers with a diameter of 1.13±0.15 μm. The total wall thickness of the R-GL/PCL was 501±35 μm (Figure 1E).

C-GL/PCL was fabricated by electrospinning GL and PCL simultaneously (Figure 1F), following the same procedure of R-GL/PCL for the inner layer (Figure 1G). For the outer part, the same solution of PCL was used, whereas the electrospinning was set up to obtain PCL fibers aligned in the circumferential direction. This modification aimed at improving the resistance to internal blood pressure. Analyses showed a high fiber alignment, with a diameter of 1.02±0.13 μm. The resulting polymeric conduit was composed of cylindrical nonwoven GL and PCL fibers surrounded by a second layer of PCL fibers circumferentially aligned (Figure 1H) with an inner diameter of 5 mm and overall thickness of 480±23 μm (Figure 1I–K). The established protocol produced a polymeric multi-layer conduit (intima-like and basal membrane-like) showing no episodes of delamination, as assessed by optical microscopy (Figure 1L).

### 2.2 Polymeric Conduit Mechanical Characterization

**Figure 2:**
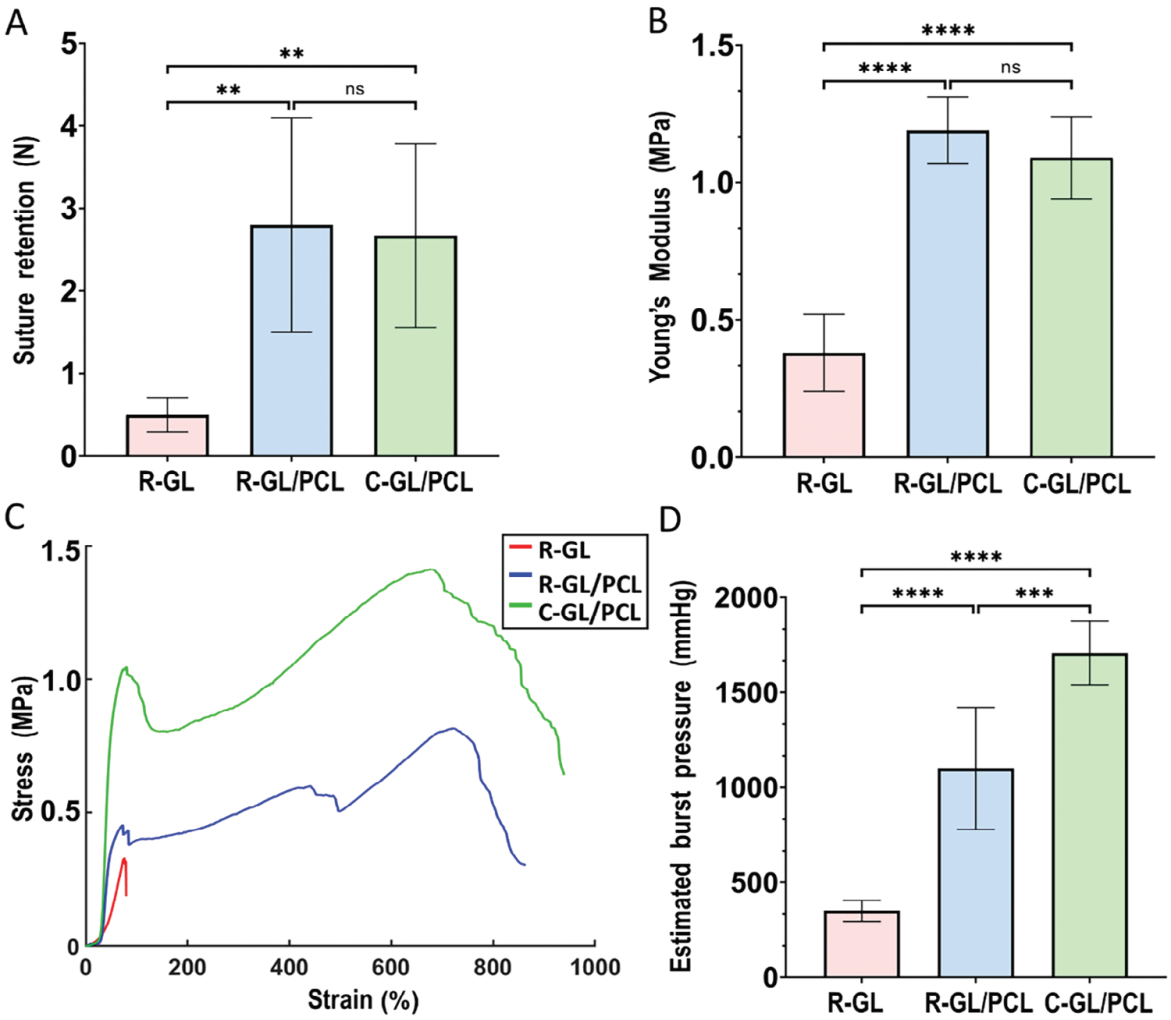
Mechanical characterization. Summary of mechanical test results on R-GL, R-GL/PCL, and C-GL/PCL conduits. A) Suture retention test, B) Young’s modulus, C) stress-strain curve of circumferential mechanical test, and D) Estimated Burst Pressure. Values are means ± SD, *N* = 6 independent replicates, each with three technical replicates. *p < 0.05, **p < 0.01, ***p < 0.001.

We performed quality tests for suture retention and mechanical resistance on the three prototypes following ISO 7198:2016. The R-GL conduit showed a suture retention strength of 0.5±0.21 N. Significantly higher values were recorded when testing the R-GL/PCL (2.8±1.3 N, *p* = 0.0031) and C-GL/PCL conduits (2.67±1.11 N, *p* = 0.0049) (number of repeats are shown in figure legends and experimental section). These data are consistent with those previously reported by Konig et al.,^[34]^ and demonstrate that the addition of PCL remarkably improved the mechanical strength of GL L (Figure 2A).

Likewise, Young’s Modulus of the R-GL evaluated in the axial direction (0.38±0.14 MPa) was significantly lower than R-GL/PCL and C-GL/PCL, which instead had comparable values (respectively 1.19±0.15 MPa (*p*<0.0001) and 1.09±0.12 MPa (*p*<0.0001)). Young’s Modulus of R-GL/PCL and C-GL/PCL were similar to that of the human coronary artery (1.48±0.24 MPa)^[35]^ (Figure 2B).

Tensile tests evaluating the circumferential stress assessed the estimated Burst Pressure (EBP) (Figure 2C).^[36,37]^ The C-GL/PCL had significantly higher EBP values (1708±167 mmHg) than the R-GL (350±55 mmHg, p<0.0001) and R-GL/PCL (1098±321 mmHg, *p*<0.0005) (Figure 2D). Importantly, values recorded on the C-GL/PCL conduit were very similar to the human SV’s reported EBP (1700 mmHg).^[17]^

Overall, the C-GL/PCL tubular scaffold had the highest mechanical performances among the multi-material/multi-scale prototypes, showing properties like native vascular tissues. Therefore, it was selected as the polymeric core for the subsequent manufacture of a complete BVM graft.

### 2.3 Graft Cellularization

We next developed a strategy for the topographic deposition of vascular cells within the BVM’s specific compartments. Two external layers (media- and adventitia-like) were created using a 3D bioprinting system, then depositing an endothelial lining facing the lumen through a rotating bioreactor.

#### 2.3.1 Muscularization

**Figure 3:**
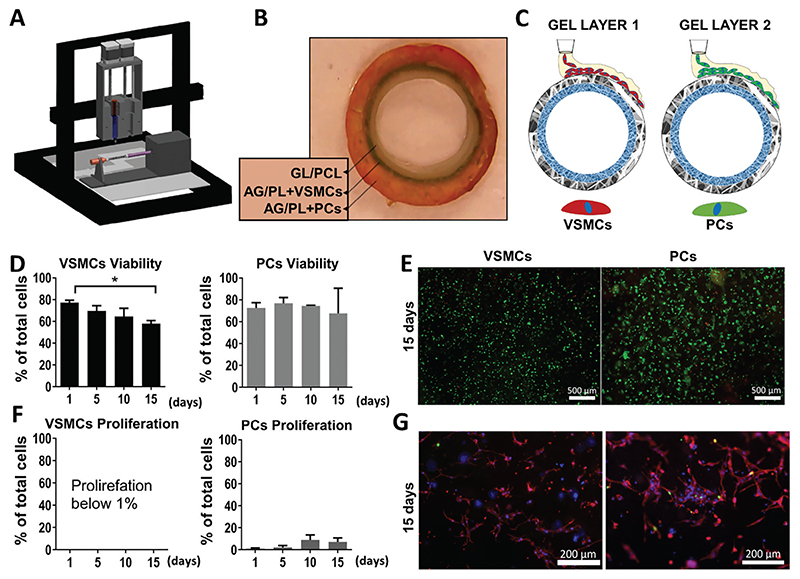
Muscularization of the BVM through a bioprinting system. Bioprinting for external cellularization. A) Schematic render of the piston-driven bioprinter and graft rotating support. B) Representative image of the C-GL/PCL polymeric structure surrounded by two concentric bioprinted AG/PL gel layers. C) Schematic representation of the two-stages bioprinting cellularization. D-E) Bar graphs show the percentage of viable PCs and VSMCs D). Representative fluorescent images of VSMCs and PCs stained with Calcein (green) and EthD-III (red) in the gel after 15 days E). F,G) Bar graphs show the percentage of proliferating PCs and VSMCs (F). Representative fluorescent images of VSMCs and PCs stained with Vimentin (red), EdU (green), and Dapi (blue) in the gel after 15 days G). Data were collected at 0, 5, 10, and 15 days of maturation in static conditions. Values are means ± SD, *N* = 3 biological replicates, each with three technical replicates. *p < 0.05.

The production of the outer layers of the vascular graft was carried out using a custom-made piston-syringe bioprinter, assisted by a rotating support for the graft (Figure 3A). To test the feasibility of the manufacturing process and the structure’s integrity, two concentric layers AG/PL gel, initially without cells, were bioprinted around the external surface of C-GL/PCL conduit.

In brief, the internal layer of extruded AG/PL was crosslinked with 20 mM calcium chloride (CaCl_2_) DMEM solution (incubation of 1 min), then the AG/PL layer was bioprinted and crosslinked with 100 mM CaCl_2_ solution (incubation of 10 min). Optical microscopy observations verified the repeatability of the procedure and confirmed that the different layers (dyed in blue and orange) had firmly attached to each other and to the underlying C-GL/PCL structure (Figure 3B).

Tuning of the crosslinking procedure showed that the 100 mM CaCl_2_ solution was optimal in terms of preserving cell viability. Once we fixed the optimal parameters for gel crosslinking, we assessed cell viability and proliferation after gel printing onto the tubular conduit.

The bioprinting procedure was first performed by seeding only one cell type at a time (first VSMCs and then PCs) (Figure 3C) and evaluating cell behavior in parallel experiments at different time points (from 1 to 15 days). The cell-laden gel was bioprinted on the external layer of the C-GL/PCL tubular structure connected to the bioprinting rotating support and crosslinked as described above. Using a dedicated culture media, the cellularized scaffold was kept tubular and cultured in static conditions in Petri dishes. The proliferation of PCs and VSMCs in the C-GL/PCL-AG/PL assembled scaffold was evaluated at the fixed times. Analysis of fluorescence microscopy images showed that PCs maintained a viability rate of around 70% over the 15-day window, while VSMCs viability decreased from 78±2% (day 1) to 57±3% (day 15) (Figure 3D,E). As assessed by Edu staining, PCs showed moderate proliferative activity at the 15-day evaluation time point. On the other hand, VSMCs showed a proliferative capacity below 1% (Figure 3F). Additional staining with Vimentin was performed on the cells stained with EdU to assess the morphology and the spreading of the cell cytoskeleton (Figure 3G). Fluorescent microscopy analysis showed that, over 15 days, PCs and VSMCs had spread, developing physiologic physical contacts within the respective layers.

#### 2.3.2 Endothelialization

**Figure 4:**
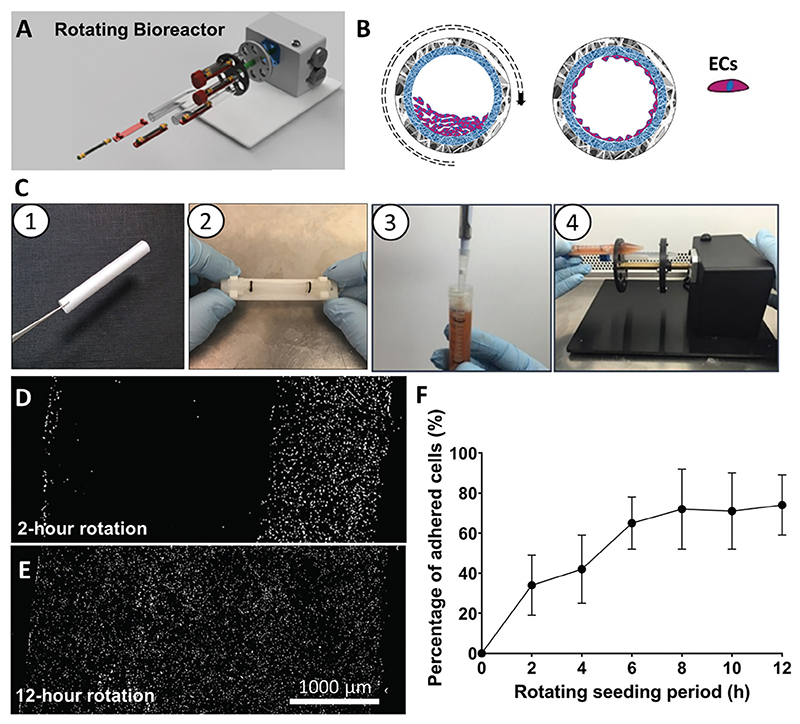
Endothelialization of the BVM carried out by rotating seeding. A,B) 3D render of the rotating bioreactor A) and the schematic stages of the cellularization B). C) Representative images of the rotative process: the sterilized tubular graft (C1) is fixed on the graft holder (C2), cells are injected into the lumen of the graft (C3), and the whole falcon tube is inserted into the rotating bioreactor shafts (C4). D,E) Representative images of ECs in the polymeric conduit lumen taken 2 h D) and 12 h from seeding E). Graph showing the percentage of the adhered ECs at different time points. *N* = 3 biological replicates, each one repeated in 3 technical replicates. G) Bar graph showing results of viability assay on ECs. H,I) Calcein (green) and EthD-1 (red) were used to stain, respectively, living, and dead cells on the control (24-well plate) I) and GL lumen of the scaffold L). J) Histology image of the EC cytoskeleton stained with vimentin (red) in the GL/PCL scaffolds lumen. K,L) magnified images of the cellularized lumen showing of ECs stained with CD31 (green) and DAPI (blue) (K), and Vimentin L). M,N) Fluorescent images of the luminal surface of the BVM conduit with ECs stained with CD31 (green) and DAPI (blue) M), and Vimentin N). *N* = 3 biological replicates, each with three technical replicates.

This process was carried out using a custom-made rotating bioreactor that enables the distribution of the cells in the conduit’s luminal surface, maintaining the whole structure in a controlled environment (Figure 4A,B).

Under sterile conditions, the C-GL/PCL conduit was locked into the holder inside the Falcon tube containing EGM-2 and 1% Penicillin/Streptomycin (P/S) and then filled with a suspension of human ECs (15 × 10^4^ cells in 400 μL culturing media) (Figure 4C). The Falcon tube containing the graft was then locked in the dedicated slots of the bioreactor. The duration of rotation was titrated to optimize the efficiency and homogeneity of the cell seeding. Fluorescent immunostaining monitored the EC coating (Figure 4D,E). This reached a plateau after 6 h (65±13% at 6-h rotation and 74±15% at 12-h rotation) (Figure 4F). Based on these data, 6 hours were chosen as a rotation time to maximize the balance between the time and efficiency of seeding.

**Figure 5:**
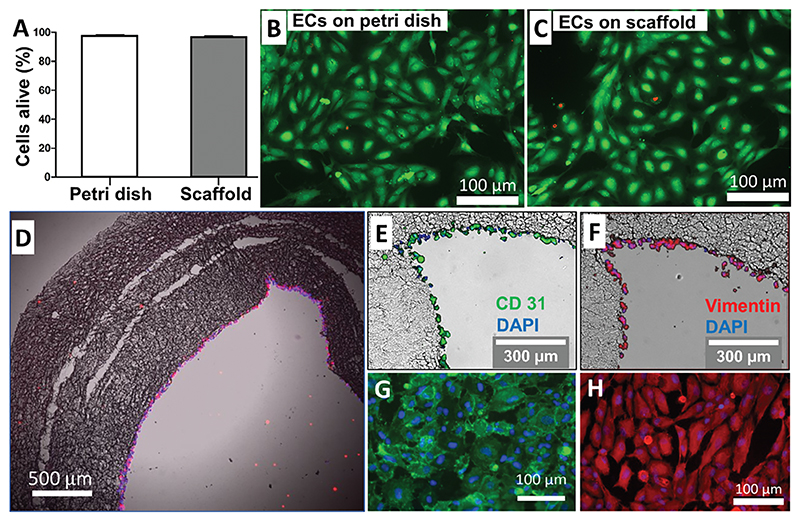
Validation of endothelialization method. A) Bar graph showing results of viability assay on ECs. B,C) Calcein (green) and EthD-1 (red) were used to stain, respectively, living, and dead cells on the control (24-well plate) B) and GL lumen of the scaffold C). D) Histology image of the EC cytoskeleton stained with vimentin (red) in the GL/PCL scaffolds lumen. K,L) magnified images of the cellularized lumen showing of ECs stained with CD31 (green) and DAPI (blue) E), and Vimentin F). M,N) Fluorescent images of the luminal surface of the BVM conduit with ECs stained with CD31 (green) and DAPI (blue) G), and Vimentin H). *N* = 3 biological replicates, each with three technical replicates.

In a parallel experiment assessing the scaffold’s biocompatibility, the conduit was transferred into Petri dishes containing EC growth media (DMEM with 10% FBS and 1% P/S), where it was cultured for 5 days (with one culture medium change). Using a Biotium viability/cytotoxicity assay kit, we could determine that 95±2% ECs in the lumen of the C-GL/PCL conduit were alive, with no difference with controls kept on plastic dishes (Figure 5A–C). Cross-sectional images captured by immunofluorescence microscopy showed a uniform layer of ECs covering the luminal surface of the conduit. No infiltration through the surrounding layer was observed (Figure 5D–H).

### 2.4 Vessel Mimic Assembly and Cultures – Semi-Automatized Assembly Line Process

**Figure 6:**
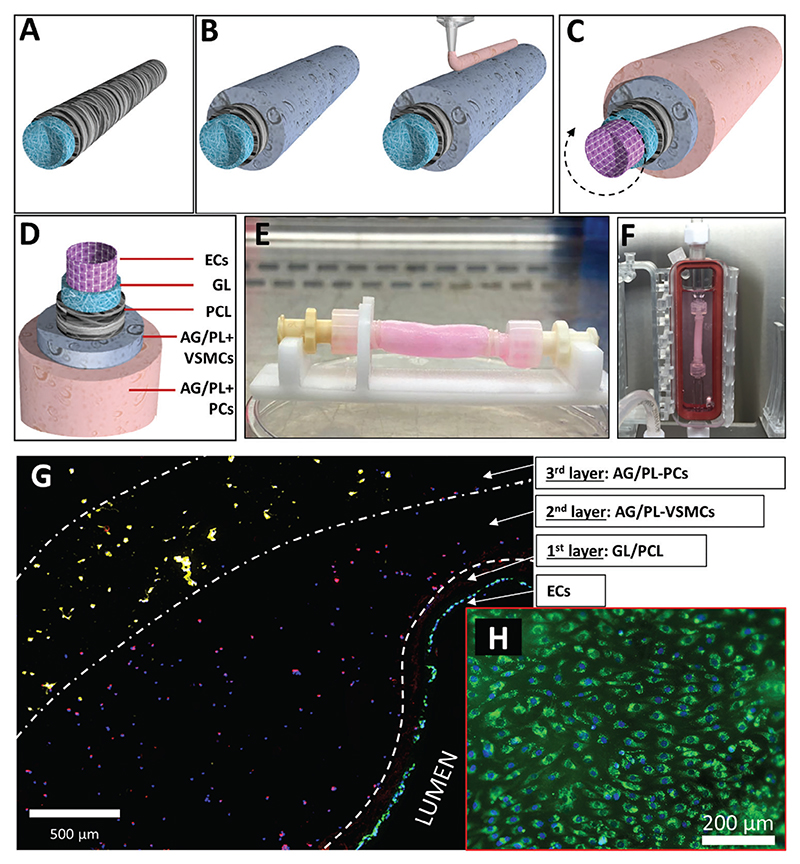
Final assembly of the BVM. A–C) Schematic 3D renders of the BVM showing the sequence of the assembly procedure: polymeric conduit fabrication (A), followed by the two-stages bioprinting of VSMC-AG/PL and PC-AG/PL B), and rotative EC seeding of C). D) Schematic 3D render of the whole BVM structure. E) Picture of the conduit after the cellularization process. F) Conduit in the bioreactor chamber for maturation under dynamic perfusion. G) Fluorescence image of the conduit cross-section showing the tri-layered structure. H) Fluorescence image of the EC lining facing the lumen.

After optimizing the seeding procedures, the protocol for producing the multi-layered BVM was finally established. The approach consisted of using the C-GL/PCL polymeric conduit as a support substrate (Figure 6A), performing the two-stage bioprinting of the external layers (Figure 6B), and completing the BVM with the EC seeding through rotation (Figure 6C,D).

Briefly, the electrospun C-GL/PCL core was cut into a 50 mm-long and 5 mm-inner diameter conduit, sterilized, and mounted on the dedicated support to perform the two-stage bioprinting of VSMC- and PC-loaded AG/PL solutions (both at the concentration of 4 million cells mL^−1^). After cross-linking the AG/PL gel, ECs were injected into the inner lumen (45 × 10^4^ cells in 400 μL of culture media) and inserted in the rotating bioreactor. Finally, the cellularized conduit (C-GL/PCL-AG/PL) (Figure 6E) underwent a 6-hour rotation followed by 5-day perfusion in the dynamic bioreactor (Figure 6F). At the end of the perfusion, shape maintenance and integrity of the structure were confirmed by visual observation and optical microscopy imaging. The persistence of the PCs (NG2, yellow dye) and VSMCs (*α*SMA, red dye) within the different compartments of the conduit was demonstrated by immunofluorescent staining (Figure 6G). ECs (von Willebrand factor (vWF), green dye) covered the whole luminal layer and did not invade the underneath C-GL/PCL layer, thus confirming the proper tuning of GL fibers (Figure 6H). Altogether, these results evidenced the feasibility of the fabrication of the multi-layered blood vessel mimic, with successful compartmentalization of cells according to a physiological topography.

### 2.5 Vessel Mimic In Vitro Hemocompatibility

Hemocompatibility was assessed in vitro by determining platelet adhesion to the luminal surface of the assembled graft. Grafts with and without endothelium were exposed to platelet-rich-plasma (PRP), and SEM images were used for quantitative and morphologic assessment of the platelet activation status.

**Figure 7:**
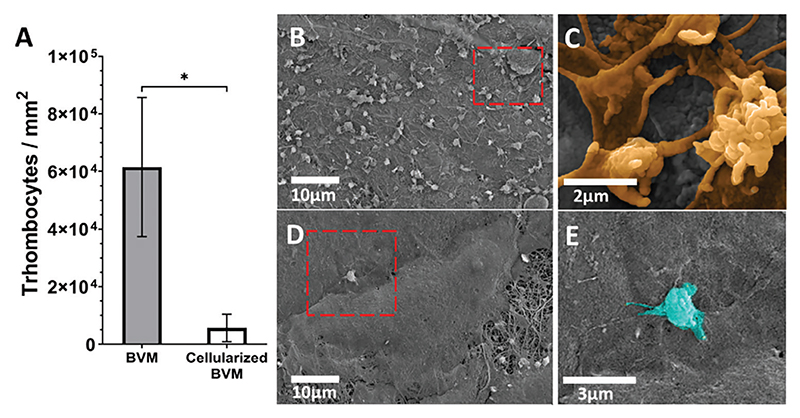
Hemocompatibility assessment. A) Bar graph illustrating data from the platelet adhesion assay. B-E) SEM images of the BVM without endothelium. B,C) and with endothelium D,E) after 1 h incubation with PRP. Images at different magnification show the activated (pseudopodia with sporuts) and fully activated (spreaded) (highlighted in orange in panel D) platelets in direct contact with the graft fibers. In contrast, ECs guarantee a minimal platelet adhesion and mostly in pseudopodia (low activation state) and inactivated state (round shape, highlighted in light blue in panel E). Values are means ± SD, *N* = 3 biological replicates, each with three technical replicates. *p = 0.0171.

Images showed a significantly lower number of adherent platelets on the cellularized BVM than on the non-cellularized one (*p* = 0.0171) (Figure 7A). In addition, SEM revealed that platelets on the graft lacking endothelium had more pseudopodia and spread morphology (associated with high activation state), compatible with an activated state (Figure 7B,C). In contrast, in the presence of the endothelium, platelets appeared to be dispersed and mostly in pseudopodia (associated with low activation state) or round shape (associated with inactivated state) (Figure 7D,E).

### 2.6 In Vivo Results

**Figure 8:**
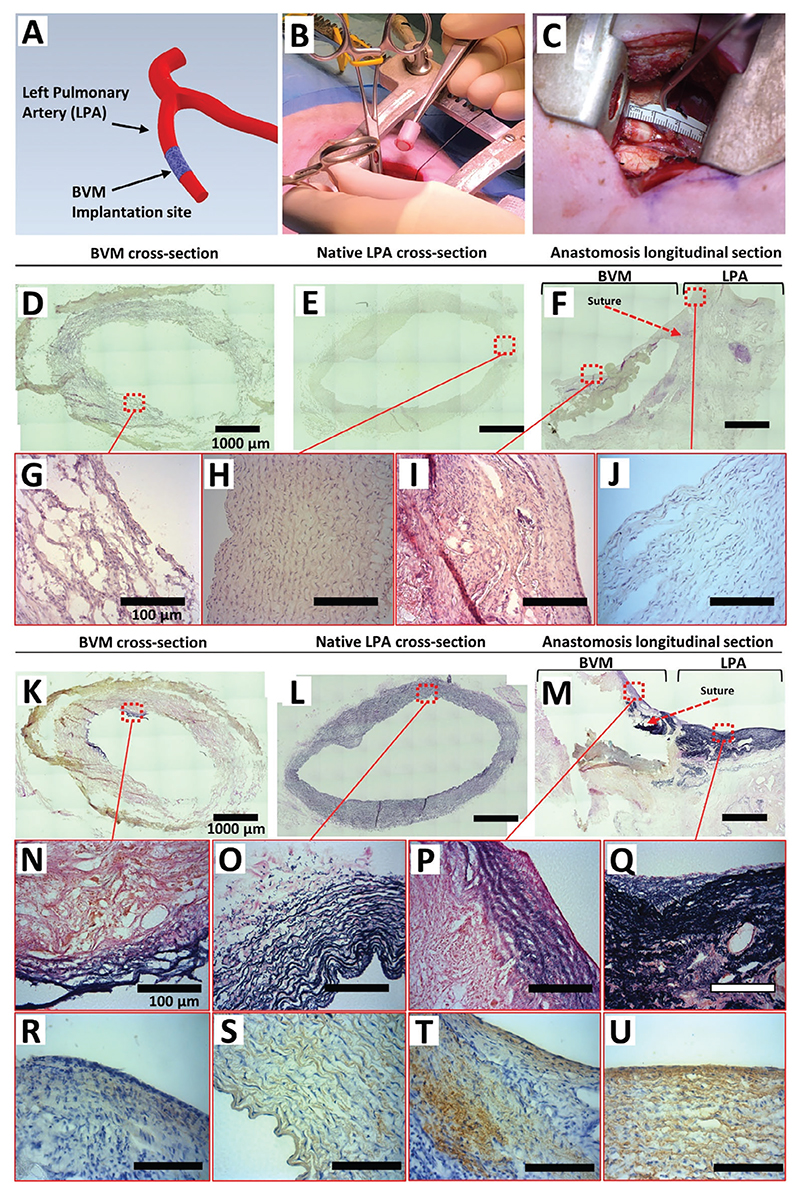
In vivo study. A) Schematic anatomic representation of the main pulmonary artery and major branches, with graft implantation area highlighted in orange. B,C) Representative images of the surgical procedures. D–F) H&E staining. Tiled Images of the explanted tissue showing the cross-section of the BVM D), native LPA E), and the longitudinal section at the anastomosis F). G–J) H&E staining. Magnified fields of cross-sections of the BVM G) and LPA H); I,J) H&E staining. Magnified fields of longitudinal sections of the BVM (I) and LPA J). K–M) EVG staining. Tiled images of the explanted tissue showing the cross-section of the BVM K), native LPA L), and the longitudinal section at the anastomosis M). N–Q) EVG staining. Magnified fields of cross-sections of the BVM N) and LPA (O); P,Q) EVG staining. Magnified fields of longitudinal sections of the BVM P) and LPA Q). R,S) Elastin staining. Magnified fields of cross-sections of the BVM R) and LPA S); T,U) Elastin staining. Magnified fields of longitudinal sections of the BVM T) and LPA U) (Brown = Elastin positive fibers).

As reported in the previous paragraph, a fully cellularized 5-mm diameter BVMs was produced and implanted in a 9-week-old Landrace female piglet under immunosuppression protocol. The BVM graft was anastomosed as an interposition graft, to the LPA, of matching internal diameter (≈5 mm) (Figure 8A).

The surgeons could easily handle the BVM graft during the procedures (Figure 8B,C). In addition, good suturability and hemostatic properties were observed, with no leakages from the graft walls, in agreement with the results from the mechanical bench tests (Video S1, Supporting Information). Patency was confirmed at 5 weeks, along with integration with the host tissue. Histological analysis showed the formation of a neointima that reduced the diameter of the conduit to 4 mm. In addition, infiltration of fibrin-like material was found between the graft and the connective tissue. Images of hematoxylin and eosin (H&E) staining showed extensive nucleation throughout the whole thickness of the structure and in both the proximal and distal parts of the graft (Figure 8D–J). Elastin is a significant component of the pulmonary artery. In line with this, elastin van Gieson (EVG) staining displayed a consistent layer of organized elastic fibers in the native LPA tissue (Figure 8K–M). Albeit in a lower quantity compared to the LPA, elastic fiber deposits were visible where the graft was sutured to native tissue, at both ends, proximal and distal (Figure 8N–Q). Specific detection of elastin showed that the amount of elastin fibers was lower at the center of the graft (equidistant from both anastomoses) compared to the LPA control (Figure 8R,S). On the other hand, elastin was present in higher amounts at the level of the anastomosis in both the graft and LPA stump (Figure 8T,U). However, image analysis showed a lower degree of organization of the elastin fibers in the graft side of the anastomosis.

**Figure 9:**
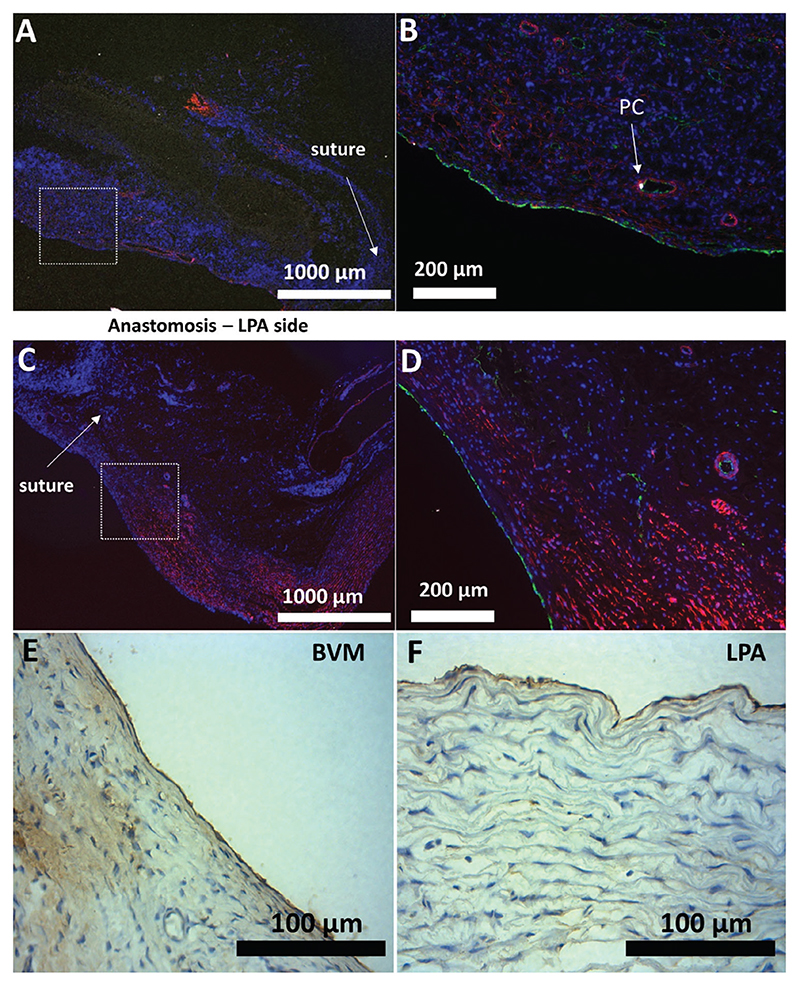
Immunofluorescence images of the harvested graft. A,B) Fluorescent images were taken on a longitudinal section of the anastomotic area (BVM side) A) and a magnified image showing the endothelialized lumen B). C,D) Fluorescent images were taken on a longitudinal section of the anastomotic area (LPA side) C) and magnified image D). Green = ECs (CD31-positive cells); Red = VSMCs (*α*-SMA-positive cells); White = PCs (NG-2-positive cells). E,F) eNOS staining; Magnified fields of cross-sections of the BVM E) and LPA F) (Brown = eNOS positive cells).

The immunohistochemical analysis identified CD31-positive ECs covering the luminal side of the LPA and implanted BMV. The LPA tunica media displayed an organized multilayer of *α*-SMA-positive VSMCs, while *α*-SMA staining was less intense at the level of the BVM (Figure 9A–D). The area of the implanted graft underneath the endothelial layer showed a more disorganized connective tissue, thus confirming what was observed in the EVG staining. It also contained an abundant microvasculature, with cells expressing NG2, likely pericytes, at the level of the adventitia. Sample sections were stained to detect human cells seeded in the BVM before implantation. Still, results from analysis of ku80 and human nuclei staining showed no residual presence of donor cells (data not shown).

Additionally, the expression of endothelial nitric oxide synthase (eNOS) by ECs of the luminal layer and intraparietal microvessels of the LPA stump and explanted graft (Figure 9E,F) was assessed by immunohistochemistry analysis. Results indicate that eNOS was expressed in ECs of both the graft and the LPA stump.

## 3 Discussion

This study reports the successful manufacture of a small diameter (<6 mm) hybrid TEVG, named BVM, that reproduces the structural complexity, mechanical properties, and cellular hierarchy of a native human artery. The two components consisting of a polymeric scaffold and cells could be stored separately and assembled to form the final product ready for implantation in one week. The BVM was suited to surgical handling and suture, showing no leakage immediately after implantation. The pilot in vivo study in swine showed good hemostatic properties, capability to withstand blood flow and patency of the BVM several weeks after implantation in the LPA.

The development of biodegradable artificial TEVG has been investigated since before the 1980s, exploring many natural,^[38–40]^ synthetic,^[41–44]^ hybrid,^[24,45–47]^ scaffold-free approaches,^[48–50]^ resulting in some clinical applications.^[51–54]^ Several novelties distinguish our BVM from previous TEVG. We used electrospinning to fabricate and compare three prototypes that differ in fiber composition (R-GL is made of GL nanofibers, while R- and C-GL/PCL have additional fibers of PCL) and organization (R-GL and R-GL/PCL have randomly organized fibers, while C-GL/PCL has an extra layer of circumferentially aligned PCL fibers). The R-GL/CPL and C-GL/PCL are based on a hybrid approach aiming to synergize the cell interaction features of GL, a natural material with high biocompatibility and cell adhesion properties, with the mechanical properties of the PCL. As expected, R-GL/PCL and C-GL/PCL showed higher Young’s modulus values and better suture retention than the R-GL. Moreover, C-GL/PCL was superior to all the other prototypes regarding EBP thanks to its layer of circumferentially aligned PCL fibers. The resulting C-GL/PCL demonstrated to match the mechanical properties of autologous vessels with Young’s modulus value comparable to the native coronary artery (1.48±0.24 MPa)^[35]^ and EBP similar to the SV (1700 mmHg).^[17]^ The intima-like layer was fabricated with co-electrospinning to create an interconnected mesh of GL and PCL fibers. This resulted in a nanofiber network with adhesive features similar to the natural extracellular matrix.^[55]^ The fiber’s dimensions and pore size were designed to allow proper nutrient exchange and ECs adhesion.^[56]^

Another significant novelty is demonstrating that the selected scaffold composition can accommodate human vascular cells to create living tissue. Previous studies attempted to replicate the complex architecture of the native artery, using innovative methodologies to produce hierarchical physical support. However, the main focus was on in situ functionalization of the graft by the recipient’s cells. A bi-layered vascular graft, obtained from overlapping different layers of fibers oriented circumferentially and transversally, was shown to improve the burst pressure of the graft.^[23]^ In another study, a hierarchically bilayered tubular scaffold comprising an inner layer of randomly oriented dense fiber mesh and an outer layer of microfibers with controlled orientation was shown to possess improved mechanical properties and to be able to direct VSMC growth in the external layer.^[27]^ Acellular scaffolds have the advantage of constituting an off-the-shelf product readily available for clinical use.^[42,57–65]^ However, the absence of the cellular component introduces some significant drawbacks: i) the time for the endogenous endothelium to spread and cover the implanted graft exposes the patient to the risk of thrombogenic events; and ii) the resistance to mechanical stress depends exclusively on the artificial graft strength, which, being intrinsically unable to remodel, could lead to aneurysm formations.

In this regard, pre-cellularized grafts present some potential advantages.^[57]^ Early efforts to build cellularized TEVGs were based on rolling confluent VSMCs and fibroblast sheets onto a rod, the so-called “cell-sheet method”, to create an artery-resembling vessel.^[49]^ The fabrication process lasted 7–9 months, and the reproducibility of the layer thickness was poor.^[16,66]^

Currently, production time and unrealistic structural/functional properties compared to native arteries represent persistent obstacles in creating successful TEVG.^[20,45,67]^

The above limitations urged the development of new methods, including seeding cells onto bio-degradable scaffolds. EC-seeded TEVGs reportedly reduce the risk of intravascular clot formation and neointima hyperplasia.^[68–70]^ VSMCs confer tonicity and elasticity due to their contractile phenotype and ability to produce ECM proteins, such as collagen and elastin.^[71]^ Moreover, the cross-talk between VSMCs and ECs is fundamental for homeostasis of the blood vessel wall, regulating ECM production and remodeling.^[72–76]^ Our three-cellular BVM surpasses previous TEVGs, which contained a maximum of two vascular cell types.^[24,77–82]^ This is the first time that PCs were included in the cellularization of a TEVG. These mural cells are typically resident in the vasculogenic zone of the adventitia, where they stabilize and regulate the functions of the vasa vasorum. Together with resident fibroblasts, they produce extracellular matrix proteins that are key for maintaining vessel structure and function.^[83]^ Moreover, PCs have important regenerative^[84]^ but also control remodeling processes in the media and intima.^[85,86]^

Combining two cellularization techniques, bioprinting and rotative deposition, allowed us to control the homogeneous seeding of different cells within the BVM layers and thus address the difficulties associated with a cylindrical structure. The semi-automatized bioprinting system achieved a precise homing of the AG/PL gel-embedded VSMCs and PCs in the BVM’s media- and adventitia-like layers. Post-seeding assessments showed PCs were broadly viable, while the viability of VSMCs decreased below 60% on day 15. The presence of damaged cells and cell debris is undesired as they can trigger an exuberant inflammatory response resulting in adverse remodeling and occlusion of the graft. The static culture of the cells might have been implicated in this phenomenon, and culture in dynamic conditions with continuous exchange of fresh nutrients may be considered to improve the long-term viability.

Moreover, PCs were proliferating after seeding, but VSMCs were not. VSMCs are known to shift reversibly from a quiescent, contractile phenotype to a synthetic, proliferative phenotype, the latter being associated with intima hyperplasia and restenosis. Therefore, the low proliferative rate observed in VSMCs within the BVM could be advantageous.^[87]^ After 2 weeks of static culturing, a morphological assessment of both VSMCs and PCs suggested that cells, starting from a globular shape at the gel deposition, spread their cytoskeleton and formed contacts, finding space within the gel in the degradation process. However, this remodeling process was insufficient to create a confluent layer in their respective location, suggesting that increasing the number of cells may be necessary.

A rotative bioreactor was used to perform the BVM endothelialization. The tuning of the rotation time allowed us to determine the optimal time to obtain a uniformly distributed layer of ECs facing the lumen (6 h). The viability assessment confirmed the procedure’s safety. Moreover, cell attachment efficiency was aligned with the average values found in the literature.^[88]^ The BVM was finally assembled with all its components confirming the feasibility of recreating the morphology of a native artery. After 5 days of maturation in a perfusion bioreactor, the BVM showed that all the cells were in the designated layers. The hemocompatibility test confirmed the importance of producing a graft with an anti-thrombogenic endothelium.

An additional advantage of the combined bioprinting and rotative seeding methodology is that it speeds up the manufacture of a reproducible product. In terms of production time, the first stage, from the bioprinting of the first AG/PL layer to the application of the flow, lasted less than 7 h, and an additional 5 days were required for dynamic conditioning, which is a significant advancement compared with previous reports.^[89,90]^ Moreover, our protocol improved the seeding efficiency compared with traditional manual seedings^[88,91]^ and rotational seeding.^[88]^ In their preclinical study in large animals, Ju et al. reported that the seeding period lasted 48 h, followed by a conditioning period in a pulsatile bioreactor of 7 days. In other studies, a polyglycolic-acid (PGA) scaffold was seeded with VSMCs for 24 h, while a small-diameter polyurethane (PU) graft was seeded for 12 h using rotational seeding.^[92]^ However, the process needed a post-seeding period of static culture to stabilize cells in the internal layer.^[93]^ Analysis of the data demonstrated that we achieved the goal of reducing the time of the cellularization process while maintaining efficiency in a valuable range (up to 75%) and consistent sterility. Moreover, the increase in technological complexity was moderated by introducing operator-friendly procedures. As a result, the newly fabricated rotating bioreactor and rotating bioprinting device allowed the seeding of cells to be carried out by a single investigator, performing all the protocol steps under a biological safety hood in sterile conditions. A recent study showed a promising automated graft fabrication system based on the simultaneous deposition of polymeric fibers and a cell-laden hydrogel that accelerates production.^[28]^ Although this TEVG demonstrated good hemocompatibility and carotid artery implantability in two rabbits, mechanical properties were not ideal. Finally, we tested the feasibility and safety of BVM implantation as LPA interposition in a piglet. The BVM showed promising features in terms of surgical manipulation, suture retention, and leak-proof blood grafting. Patency after 5 weeks was confirmed in the LPA interposition model, along with the ability of the BVM to match the native artery growth. The histology data indicated that the BVM underwent a remodeling process with evidence of cellularization (including a layer of eNOS-positive ECs) and elastin deposition. However, elastin deposition was mainly localized in the anastomotic areas of the graft which suggests the need of longer time of implantation for complete elastin deposition along the whole length of the graft. There was no signal for human cells in the graft, suggesting they were removed by the host’s immune system response despite the immunosuppressive treatment and substituted by endogenous cells. These results support using a full swine-to-swine approach in a future efficacy study.

## 4 Conclusions

In conclusion, this study presented the production of a novel small-diameter BVM, which may constitute a solution to the lack of autologous vessels in cardiovascular surgery. Future preclinical work will focus on using swine cells to decorate the BVM, followed by allogenic implantation in a swine model. Regarding potential commercial barriers toward clinical applications, the BVM falls into the tissue-engineered and advanced therapy medicinal products (ATMPs) category. Hence, it is likely to classify BVM as a “combined tissue-engineered product (TEP)” by regulatory agencies. The superiority in efficacy could balance the high cost of production. We envisage the BVM application limited to patients with vascular disease who require distal bypass surgery for limb salvage, in whom an autologous conduit is unavailable, and other established treatments, such as percutaneous angioplasty, have been exhausted.

## 5 Experimental Section

### Ethics

Institutional review board approval for the study was obtained according to the guidelines noted in the Journal Instructions to Authors. Animal experiments were performed according to institutional guidelines. They followed the principles stated in the Guide for Care and Use of Laboratory Animals published by the National Institutes of Health in 1996 and the Animals (Scientific Procedures) Act published in 1986. The protocol was covered by the UK Home Office ethical approval PPL 30/3019 and PF6E6335D. The report of experimental data follows the Animal Research: Reporting of In Vivo Experiments (ARRIVE) guidelines.^[94]^

### Polymeric Conduit Fabrication

The Electrospinning techniques were used to fabricate the three prototypes of the innermost layer of the graft:

i) R-GL; ii) R-GL/PCL; iii) C-GL/PCL.

R-GL consists of a polymeric conduit entirely formed of GL nanofibers. Before electrospinning, a solution of GL 15% (w v^−1^) (Type A gelatin from porcine skin, Sigma-Aldrich) was prepared by dissolving GL powder in a liquid solution of 60/40 (v v^−1^) acetic acid/deionized water. A crosslinking agent GPTMS (Sigma-Aldrich) was added at the concentration of 3% (v v^−1^) and stirred for 1 h before using the solution. The GL solution was electrospun on the 5-mm diameter mandrel rotating at 1000 rpm for 3 h, and the electrospinning device (Electrospinning Station, Nadetech, Navarra Spain) was set as 20 kV (Voltage), 15 cm (distance from the collector) and 0.2 mL hour^−1^ (flow rate). After the deposition. The conduit was dried at 37 °C for 48 h to allow the GL crosslinking.

To generate the second prototype R-GL/PCL, two subsequent steps of electrospinning deposition were performed on the cylindrical rotating collector: i) co-electrospinning of GL and PCL solutions; ii) electrospinning of PCL solution alone. The solution of 15% (w v^−1^) GL was prepared following the same procedure of R-GL. PCL (Mn average 80000, Sigma-Aldrich) was dissolved in Chloroform to obtain a 15% (w v^−1^) solution. For the inner layer, two distinct 1-mL syringes were filled with GL or PCL solution and connected to two 21 G blunt-ended needles that served as the charged spinneret. The two solutions were simultaneously electrospun on the mandrel rotating at 1000 rpm for 1 h, and the electrospinning device was set as 20 kV (Voltage), 12 cm (distance from the collector), and 0.2 mL hour^−1^ (flow rate). For the external layer, 15% (w v^−1^) PCL solution was electrospun on the rotating mandrel (1000 rpm) at 12 kV, 12 cm, and 0.5 mL hour^−1^. After 2 h of deposition, the conduit was dried at 37 °C for 48 h.

C-GL/PCL conduit production protocol saw the first stage of co-electrospinning of GL and PCL solutions, followed by an electrospinning deposition of aligned PCL fibers. GL and PCL co-electrospinning followed the same procedure of the R-GL/PCL, meaning 15% (w v^−1^) GL and 15% (w v^−1^) PCL were electrospun for 1 h at 20 kV (Voltage), 12 cm (distance from the collector), 0.2 mL hour^−1^ (flow rate), and 1000 rpm. For the external layer, the same PCL solution was electrospun on the mandrel rotating at 6000 rpm, 12 kV, 12 cm, and 0.5 mL hour^−1^. Also, in this case, the conduit was dried at 37°C for 48 hours to allow the crosslinking of GL.

The tubular scaffolds were stored at room temperature in dry conditions before characterization and use for cell culture.

### Morphological Characterization

The scaffold’s inner and outer surfaces were sputter coated with an 8 nm platinum coating (EM ACE600, Leica) and analyzed via SEM (Crossbeam 340, Carl Zeiss). The diameter of the electrospun fibers was measured based on the images using ImageJ at ten different locations, respectively. The porosity of the electrospun non-woven meshes was analyzed and processed using ImageJ to calculate the porosity of the scaffolds.

### Mechanical Testing

R-GL, R-GL/PCL, and C-GL/PCL polymeric conduits were tested in uniaxial tension using a universal testing machine (Instron 3342, Norwood, MA, USA) with a 100 N load cell. Longitudinal, circumferential, and suture retention tests were carried out per ISO 7198:2016.

For the longitudinal test, the scaffolds were cut into 10 × 65 mm (length x width) stripes, maintained, and tested under in vitro conditions by immersion in PBS at 37 ± 0.5°C. Sample thickness and width were measured using a micrometer. Uniaxial tensile testing was performed at a constant strain rate of 50 mm min^−1^ and up to rupture. Stress-strain curves for all experiments were obtained from axial loading and clamp displacement data recorded during the test. Stress was computed as F/A, where the F corresponds to the tensile load with a precision of 0.01 N and A is the initial cross-sectional area. The strain was calculated as 100 × L/L0, with L and L0 as the current and initial sample length.

Tubular structures were cut in rings to perform circumferential tensile tests. The 10mm-wide rings were placed onto two pins and stretched at a uniform rate of 50 mm min^−1^ until the breaking point was reached.^[95]^ The load at yield or break, the ultimate tensile stress (UTS), and extension were measured to calculate the circumferential tensile strength (*Yc*) using the equation:

(1) $$Y_{c}=\frac{UTS}{2L}$$

where L is the original length of the sample.

Burst pressure was estimated from UTS using the law of Laplace, as described by Nieponice et al.^[34,96,97]^

To perform suture retention strength, a 50 mm length sample obtained from a longitudinal conduit cut was mounted by clamping one end at the tensile machine, whereas the other end was sutured using polypropylene surgical suture (6-0 PROLENE, ETHICON) in one point at 2 mm from the free edge, in which a half loop formed by the suture is fixed to the other movable clamp. The pulling speed was set to 50 mm min^−1^. Load value was recorded at the time of suture failure.

All the experiments were performed on 6 independent replicates, each assessed in technical triplicates.

### Cell Sources

PCs were isolated from the SV from leftovers of surgical bypass grafting and expanded using a previously described protocol.^[98]^ Briefly, cells obtained from the digestion of the vein with 3.7 mg mL^−1^ Liberase 2 (Roche, Basel, Switzerland) went through multiple steps of immunomagnetic sorting using anti-CD31 conjugated beads, anti-CD34 beads (Miltenyi, Bergisch Gladbach, Germany). The obtained PCs (CD31-, CD34+) were cultured on plates coated with Fibronectin (10 μg mL^−1^) and gelatin (0.1%) (both from Sigma-Aldrich, Dorset, UK) in the presence of growth medium, EGM-2 (PromoCell cat#: C-22111). The PC’s antigenic phenotype was evaluated by flow cytometry using a FACS Canto II flow cytometer and FACS Diva software (BD Biosciences). A combination of the following antibodies were employed: anti-CD44 (eBioscience), anti-CD-105 (Life Technologies), and anti-CD90 (BD biosciences). The purity of the cell preparation was confirmed, with >95% of cells expressing the above markers. Studies using human cells were covered by Research Ethics Committee approvals (06/Q2001/197 and 11/2009) and complied with the principles stated in the 1964 Declaration of Helsinki and later amendments. All the subjects gave informed written consent for the experimental use of donated material (Table S1, Supporting Information).

Commercially available human ECs (PromoCell cat#: C-12221) and human VSMCs (PromoCell cat#: C-12511) were cultured at 37°C, 20% O_2_, 5% CO_2_ respectively in GM-MV2 (PromoCell, cat#: C-22121) and smooth muscle cell growth medium (SMCGM-2, Promocell, cat#: C-22162).

All in vitro and in vivo experiments were set up with PCs, ECs, and VSMCs between passages 5 and 7.

### Gel Production and Crosslinking Tuning

The AG and PL solutions were produced following the protocol described in a previous work.^[99]^ Briefly, the final hydrogel working solution was produced by combining solutions of PL and AG to achieve a final gel of 13% (w v^−1^) PL and 6% (w v^−1^) AG in serum-free DMEM (Gibco Life TechnologiesTM).

The crosslinking process of the two consecutive bioprinting steps around the external surface of the electrospun conduit was then tuned (Table S2, Supporting Information). The leakage of gel or the loss of shape of the extruded gel would lead to the failure of the manufacturing process. The solution of CaCl_2_ was used as a cross-linker for the AG/PL gel. For this test, carried out without cells, the AG/PL gel was extruded on the external surface of the C-GL/PCL tubular structure while the scaffold was rotating until all the surface was covered. The structure was incubated in a 20 mM CaCl_2_ solution for 1 min, and the second layer of gel was extruded onto the previous one to form two concentric layers. Finally, the whole complex was incubated with 100 mM CaCl_2_ solution for 10 min.

### External Cellularization – 3D Bioprinting

The novel methodology based on rotating 3D bioprinting was designed to allow the concentric extrusion of two gel layers with VSMCs and PCs.

The bioprinting of the AG/PL gel layers was carried out using a custom-made piston-syringe bioprinter (MandleMax, Maker’s Tool Works, US) previously developed in our laboratories.^[13,100]^ In addition, rotating support for the graft allowed the extrusion on the cylindrical surface of the scaffold (Figure S1, Supporting information). The rotation of the support was synchronized with the bioprinter enabling a uniform deposition of the gels. These devices were accompanied by a sterile tank that was used to incubate the graft with the CaCl_2_ solutions for crosslinking, defined as a crosslinking tank.

In separate experiments, we seeded VSMCs, or PCs in AG/PL, on the external surface of the tubular structure to allow better visualization of the success of the seeding procedure through fluorescence microscopy and assess the viability and proliferation for each cell type.

The bioprinting rotating system was sterilized with Eth 70% (v/v), exposed under UV light for at least 1 h, and kept in the biological safety cabinet in a sterile condition until the moment of use. The graft rotating support was positioned on the rotating support of the bioprinter, and the focus of the process moved to the loading of the cell in the gel.

VSMCs at passages 5–7 were counted and centrifuged to obtain a concentrated pellet. The pellet was then added into the AG/PL gel and loaded into a 5 mL sterile syringe with a density of 4 million cells mL^−1^. When the cell-laden gel was ready, the syringe was placed in the bioprinter, the C-GL/PCL scaffold was inserted into the sterile rotating rod, and the gel was extruded. Once the gel layer was concluded, it was moved into the crosslinking tank, filled with 100 mM CaCl_2_ SMCGM-2 culture media, for 10 min at 37 °C. The same procedure was performed for PCs in a parallel experiment.

Viability assay (viability/cytotoxicity assay kit, Biotium Inc, US) was performed at 1, 5, 10, and 15 days to evaluate the trend of cell viability in the gel. Same time points were also fixed to assess the proliferation of cells (Click-iT EdU Assay, Invitrogen). All experiments were performed on three biological replicates, each evaluated in technical triplicates.

### Lumen Cellularization

A rotating bioreactor was used to achieve the homogeneous seeding of the graft lumen. It consists of a custom-made rotating device that sees the controlled motor system connected to a carrier able to accommodate 6 Falcon tubes where the vascular graft could be positioned (Figure S2, Supporting information). An ad-hoc graft holder made of Polyoxymethylene (POM) was designed to block the graft in position during the rotation period, avoiding impacts and possible damage to the external surface of the graft (Figure S2, Supporting information), and able to tightly fit a disposable Falcon tube. The rotating bioreactor is fully programmable and designed to be easily sterilizable with ethanol and UV, to work inside an incubator at 37 °C, and guarantee biological safety.

The rotative protocol consists of a 20-s clockwise rotation, a 30-sec anti-clockwise rotation at 13.4 rpm, followed by 5 min rest, and the user can set the numbers cycles by a programmable interface. The seeding procedure underwent a tuning of the rotating duration.

The C-GL/PCL sterilized scaffold was cut in a 50mm-long conduit, locked into the holder POM holder, and inserted into the falcon tube containing EGM-2 and 1% Penicillin/Streptomycin. ECs at passage 5–7 were suspended in culture medium solution, injected in the lumen of the graft (15 × 10^4^ cells in 400 μL culturing media), and the extremities of the graft closed with sterile polydimethylsiloxane (PDMS) cap (Sylgard 184, Sigma-Aldrich).

The rotating bioreactor was activated, and the experiment was stopped at 2, 4, 6, 8, 10, and 12 h. For each time point, the scaffold was extracted from the holder, fixed in 4% paraformaldehyde (PFA), and permeabilized in 0.1% Triton (Sigma-Aldrich). Anti-Vimentin antibody (25 μL mL^−1^ in PBS, Ab92547, Abcam) and Phalloidin (Conjugated Alexa Fluor 555 Phalloidin A34055, Life Technologies) were used as dyes for the cytoskeleton. Nuclei were stained with DAPI. The samples were observed under the fluorescence microscope Zeiss Axio observer Z1 (Zeiss). The culture media from inside the lumen of the graft and the volume in the falcon tube were collected and examined for the possible presence of cells. Once the duration tuning was accomplished, the number of cells injected was increased to 45 × 10^4^ cells in 400 μL.

In a parallel experiment, after a 6-h rotational period, the C-GL/PCL conduit seeded with ECs was moved into a petri dish for 5 days in static culture in an incubator at 37 °C, 5% CO_2_, and at the end of the culture period, the viability of ECs was assessed (viability/cytotoxicity assay kit, Biotium Inc, US). ECs cells seeded on a commercial petri dish and cultured in the same environment were used as control, and the experiment was performed in 3 biological replicates, each with 3 technical replicates.

Graft sections and luminal surface portions were incubated with CD31 (1:50, R&D system, UK) or Vimentin (1:50, Abcam, UK) to detect ECs distribution.

### Fabrication of the Complete Cellularized Graft/Vessel Mimic

The full bioinspired TEVG was finally assembled, consisting of a three-layered graft. The inner layer was composed of a C-GL/PCL nanofibers conduit manufactured by electrospinning as described above. The resulting polymeric structure was cut into the 50 mm-long conduits, sterilized (Supporting information), and inserted onto the sterile Nylon support fitted into the seeding tank.

As explained previously, AG/PL gel was prepared for the middle layer to obtain a final concentration of AG 6% (w v^−1^) and PL 13% (w v^−1^) in DMEM. VSMCs (4 million cells mL^−1^) were loaded in the AG/PL gel and added in a sterile 5 mL syringe of the bioprinting system. The VSMC-laden gel was then extruded on top of the polymeric layer while rotating. The gel was crosslinked for 1 min in a solution of 20 mM CaCl_2_ in DMEM.

Subsequently, the concentric-outer AG/PL layer containing PCs was extruded on top of the middle layer (PCs at 4 million cells mL^−1^). The final structure was crosslinked with 100 mM CaCl_2_ solution in DMEM for 10 min. The procedure was performed under sterile conditions within a biosafety cabinet, including the AG/PL hydrogel preparation.

Once the external layers of the scaffold were produced, ECs were added to complete the full cellularization. The C-GL/PCL scaffold loaded with two layers of AG/PL was pulled off from the support, mounted onto the holders of the rotating bioreactor, and placed into a 50 mL Falcon tube filled with EGM-2 with 1% (v v^−1^) penicillin–streptomycin, as described in the previous paragraph. ECS was now injected in the lumen of the three-layered scaffold (45 × 10^4^ cells in 400 μL culture media), the extremities of the graft closed with sterile caps, and the whole falcon tube was placed in the rotating bioreactor for a rotation period of 6 h.

At the end of the rotational period, the complete hybrid TEVG was moved into a 3DCulturePro Bioreactor (TA Instruments, UK) for perfusion conditioning. The TEVG was kept in the bioreactor withstanding 10 mL min^−1^ flow for 5 days, and at the end of the period, an integrity check of the multiple layers was performed, together with functional assays of the cells.

### Immunofluorescent Staining

Once removed from the perfusion bioreactor, tissue cross-sections were stained with anti-*α*-SMA (1:100, Dako, UK), NG-2 (1:50, Millipore, UK), and von Willebrand factor (vWF) (1:20, Dako, UK) to detect VSMCs, PCs, and ECs respectively. Nuclei were stained with 1:1000 (v v^−1^) DAPI solution in PBS; sections were mounted with Fluoromount G.

### In Vitro Hemocompatibility

In vitro blood compatibility assay was performed according to Fernandez-Colino et al.^[101]^ Briefly, 8 mL blood samples were collected from healthy volunteers and mixed at the volumetric ratio of 1:9 with 3.2% Sodium Citrate in BD Vacutainer (BD Diagnostics, UK). The blood samples were divided in two aliquots; four mL were centrifuged at 2000 × *g* for 15 min, while the other 4 mL were centrifuged at 250 x *g* for 15 minutes. The plasmas derived from the two blood samples were mixed at ratio of 1:1 obtaining a PRP. At the day of the experiment, samples of TEVG without cells and TEVG previously cellularized with ECs and cultured for 5 days were cut open in specimens of 10 × 10 mm and placed in 24-well plate. The TEVG samples were incubated with PRP for 1 h at 37 °C, subsequently washed with PBS and fixed with 4% PFA. Platelet adhesion on the surface was then inspected with SEM imaging. Samples were dehydrated in ethanol solutions of 30%,50%, 70%, 80%, 90%, 96%, and three times 100%, for 10 min each, followed by incubation in hexamethyldisilazane (HMDS) for 5 min. After drying, the samples were sputter coated and examined with Zeiss SEM.

### Implantation in a Pig Model

A 9-week-old littermate Landrace female piglet underwent immunosuppression therapy using cyclosporin (15 mg kg^−1^ a day) and methylprednisolone (125 mg day^−1^) 2 days before surgery up to termination. Under general anesthesia and neuromuscular blockade, the piglet underwent LPA interposition to understand the BVM capability to be handled and sutured in an end-to-end reconstruction (the surgical procedure described in detail in Supporting Information).

The piglet received a fully cellularized BVM cultured under rotating conditions (6 h) and dynamic conditions (5 days). A portion of the artery (5-6 mm internal diameter) was resected to accommodate the graft (≈10 mm long and ≈5 mm in diameter), with the same procedure previously reported.^[102]^

The animal recovered under intense postoperative monitoring for the initial 24 h. Imaging studies were performed using a 2D Doppler echocardiography system (VividQ; GE Healthcare, Cardiff, UK) at baseline. Then, the piglet was euthanized, and the grafted LPA was harvested. Tissues were snap frozen, fresh frozen in optimal cutting temperature compound, or fixed in 4% PFA before paraffin inclusion. Sections were used for histology, immunohistochemistry, and morphometric analyses.

### Histology

Surgically explanted BVMs from pig LPA and aorta models were prepared for H&E and EVG histological studies. Briefly, BVMs were fixed in 4% (w v^−1^) PFA solution overnight at 4 °C, placed in 30% (w v^−1^) sucrose, and embedded in optimal cutting temperature (OCT) compound to obtain frozen tissues. Five-μm-thick sections slides were stained for H&E and EVG, to assess the structure of the tissue and the presence of elastic fibers. Later, the samples were stained for elastin fibers and eNOS expression for quantification (more detailed description in Supporting Information).

In addition, the newly formed arterial structure was detected with immunofluorescent staining of intraparietal PCs, VSMCs, and ECs in the lumen, and to evaluate the remodeling in vivo of the TEVG detection of human cells was performed using ku80 antibody (R&D systems, UK).

### Statistical Analysis

Continuous variables normally distributed were compared using the Student’s *t*-test (two-group comparison) or one-way analysis of variance followed by Tukey PostHoc analysis (ANOVA; for multiple group comparisons), as appropriate. Analyses were performed using GraphPad Prism 8.0 statistical software.

## Supplementary Material

Supporting Information

## Data Availability

The data that support the findings of this study are available from the corresponding author upon reasonable request.

## References

[1] Townsend N, Kazakiewicz D, Lucy Wright F, Timmis A, Huculeci R, Torbica A, Gale CP, Achenbach S, Weidinger F, Vardas P (2022). Nat Rev Cardiol.

[2] Timmis A, Townsend N, Gale CP, Torbica A, Lettino M, Petersen SE, Mossialos EA, Maggioni AP, Kazakiewicz D, May HT, De Smedt D (2020). Eur Heart J.

[3] Melly L, Torregrossa G, Lee T, Jansens JL, Puskas JD (2018). J Thorac Dis.

[4] Angelini GD, Culliford L, Smith DK, Hamilton MCK, Murphy GJ, Ascione R, Baumbach A, Reeves BC (2009). J Thorac Cardiovasc Surg.

[5] Harskamp RE, Lopes RD, Baisden CE, De Winter RJ, Alexander JH (2013). Ann Surg.

[6] Chlupác J, Filová E, Bačáková L (2009). Physiol Res.

[7] Ballotta E, Renon L, Toffano M, Da Giau G (2003). J Vasc Surg.

[8] Pampaloni F, Reynaud EG, Stelzer EHK (2007). Nat Rev Mol Cell Biol.

[9] Seifu DG, Purnama A, Mequanint K, Mantovani D (2013). Nat Rev Cardiol.

[10] Shojaee M, Bashur CA (2017). Adv Healthcare Mater.

[11] Kim GH (2008). Biomed Mater.

[12] Yang G-H, Kim M, Kim G (2015). J Colloid Interface Sci.

[13] Carrabba M, Jover E, Fagnano M, Thomas AC, Avolio E, Richardson T, Carter B, Vozzi G, Perriman AW, Madeddu P (2020). Front Cardiovasc Med.

[14] Hasan A, Memic A, Annabi N, Hossain M, Paul A, Dokmeci MR, Dehghani F, Khademhosseini A (2014). Acta Biomater.

[15] Driessen NJB, Cox MAJ, Bouten CVC, Baaijens FPT (2008). Biomech Model Mechanobiol.

[16] Mcallister TN, Maruszewski M, Garrido SA, Wystrychowski W, Dusserre N, Marini A, Zagalski K, Fiorillo A, Avila H, Manglano X, Antonelli J (2009). Lancet.

[17] Deutsch M, Meinhart J, Zilla P, Howanietz N, Gorlitzer M, Froeschl A, Stuempflen A, Bezuidenhout D, Grabenwoeger M (2009). J Vasc Surg.

[18] Pashneh-Tala S, MacNeil S, Claeyssens F (2015). Tissue Eng Part B Rev.

[19] Gong W, Lei D, Li S, Huang P, Qi Q, Sun Y, Zhang Y, Wang Z, You Z, Ye X, Zhao Q (2016). Biomaterials.

[20] Carrabba M, Madeddu P (2018). Front Bioeng Biotechnol.

[21] Sarkar S, Salacinski HJ, Hamilton G, Seifalian AM (2006). Eur J Vasc Endovasc Surg.

[22] Wang Y, Hu J, Jiao J, Liu Z, Zhou Z, Zhao C, Chang L-J, Chen YE, Ma PX, Yang B (2014). Biomaterials.

[23] Zhu M, Wang Z, Zhang J, Wang L, Yang X, Chen J, Fan G, Ji S, Xing C, Wang K, Zhao Q (2015). Biomaterials.

[24] Ju YM, Ahn H, Arenas-Herrera J, Kim C, Abolbashari M, Atala A, Yoo JJ, Lee SJ (2017). Acta Biomater.

[25] Yuan B, Jin Y, Sun Y, Wang D, Sun J, Wang Z, Zhang W, Jiang X (2012). Adv Mater.

[26] Liu K, Wang N, Wang W, Shi L, Li H, Guo F, Zhang L, Kong L, Wang S, Zhao Y (2017). J Mater Chem B.

[27] Jungst T, Pennings I, Schmitz M, Rosenberg AJWP, Groll J, Gawlitta D (2019). Adv Funct Mater.

[28] Akentjew TL, Terraza C, Suazo C, Maksimuck J, Wilkens CA, Vargas F, Zavala G, Ocaña M, Enrione J, García-Herrera CM (2019). Nat Commun.

[29] Yue B (2014). J Glaucoma.

[30] Ran X, Ye Z, Fu M, Wang Q, Wu H, Lin S, Yin T, Hu T, Wang G (2019). Macromol Biosci.

[31] Tonda-Turo C, Cipriani E, Gnavi S, Chiono V, Mattu C, Gentile P, Perroteau I, Zanetti M, Ciardelli G (2013). Mater Sci Eng C.

[32] Powell HM, Boyce ST (2007). J Biomed Mater Res Part A.

[33] Lü L-X, Wang Y-Y, Mao X, Xiao Z-D, Huang N-P (2012). Biomed Mater.

[34] Konig G, Mcallister TN, Dusserre N, Garrido SA, Iyican C, Marini A, Fiorillo A, Avila H, Wystrychowski W, Zagalski K, Maruszewski M (2009). Biomaterials.

[35] Karimi A, Navidbakhsh M, Shojaei A, Faghihi S (2013). Mater Sci Eng C.

[36] Van Uden S, Vanerio N, Catto V, Bonandrini B, Tironi M, Figliuzzi M, Remuzzi A, Kock L, Redaelli ACL, Greco FG, Riboldi SA (2019). Biomed Mater.

[37] Riboldi SA, Tozzi M, Bagardi M, Ravasio G, Cigalino G, Crippa L, Piccolo S, Nahal A, Spandri M, Catto V, Tironi M (2020). Adv Healthcare Mater.

[38] Hao W, Han J, Chu Y, Huang L, Zhuang Y, Sun J, Li X, Zhao Y, Chen Y, Dai J (2018). Macromol Biosci.

[39] Filipe EC, Santos M, Hung J, Lee BSL, Yang N, Chan AHP, Ng MKC, Rnjak-Kovacina J, Wise SG (2018). JACC Basic to Transl Sci.

[40] Grus T, Lambert L, Mlcek M, Chlup H, Honsova E, Spacek M, Burgetova A, Lindner J (2018). Biomed Res Int.

[41] Athanasiou K (1996). Biomaterials.

[42] Pektok E, Nottelet B, Tille J-C, Gurny R, Kalangos A, Moeller M, Walpoth BH (2008). Circulation.

[43] Sugiura T, Tara S, Nakayama H, Kurobe H, Yi T, Lee Y-U, Lee AY, Breuer CK, Shinoka T (2016). Ann Thorac Surg.

[44] Torikai K, Ichikawa H, Hirakawa K, Matsumiya G, Kuratani T, Iwai S, Saito A, Kawaguchi N, Matsuura N, Sawa Y (2008). J Thorac Cardiovasc Surg.

[45] Koch S, Flanagan TC, Sachweh JS, Tanios F, Schnoering H, Deichmann T, Ellä V, Kellomäki M, Gronloh N, Gries T, Tolba R (2010). Biomaterials.

[46] Tillman BW, Yazdani SK, Lee SJ, Geary RL, Atala A, Yoo JJ (2009). Biomaterials.

[47] Haghjooy Javanmard S, Anari J, Zargar Kharazi A, Vatankhah E (2016). J Biomater Appl.

[48] L’Heureux N, Pâquet S, Labbé R, Germain L, Auger FA (1998). FASEB J.

[49] L’Heureux N, Dusserre N, Konig G, Victor B, Keire P, Wight TN, Chronos NAF, Kyles AE, Gregory CR, Hoyt G, Robbins RC (2006). Nat Med.

[50] Zhao J, Liu L, Wei J, Ma D, Geng W, Yan X, Zhu J, Du H, Liu Y, Li L, Chen F (2012). Artif Organs.

[51] Olausson M, Patil PB, Kuna VK, Chougule P, Hernandez N, Methe K, Kullberg-Lindh C, Borg H, Ejnell H, Sumitran-Holgersson S (2012). Lancet.

[52] Lindsey P, Echeverria A, Cheung M, Kfoury E, Bechara CF, Lin PH (2018). World J Surg.

[53] Lawson JH, Glickman MH, Ilzecki M, Jakimowicz T, Jaroszynski A, Peden EK, Pilgrim AJ, Prichard HL, Guziewicz M, Przywara S, Szmidt J (2016). Lancet.

[54] Wystrychowski W, Mcallister TN, Zagalski K, Dusserre N, Cierpka L, L’heureux N (2014). J Vasc Surg.

[55] Carrabba M, De Maria C, Oikawa A, Reni C, Rodriguez-Arabaolaza I, Spencer H, Slater S, Avolio E, Dang Z, Spinetti G, Madeddu P (2016). Biofabrication.

[56] Powell HM, Boyce ST (2008). J Biomed Mater Res – Part A.

[57] Stowell CET, Wang Y (2018). Biomaterials.

[58] Mahara A, Somekawa S, Kobayashi N, Hirano Y, Kimura Y, Fujisato T, Yamaoka T (2015). Biomaterials.

[59] Shafiq M, Jung Y, Kim SH (2016). J Biomed Mater Res Part A.

[60] Muylaert DEP, Van Almen GC, Talacua H, Fledderus JO, Kluin J, Hendrikse SIS, Van Dongen JLJ, Sijbesma E, Bosman AW, Mes T, Thakkar SH (2016). Biomaterials.

[61] Wissing TB, Bonito V, Bouten CVC, Smits AIPM (2017). npj Regen Med.

[62] Valence SD, Tille J-C, Chaabane C, Gurny R, Bochaton-Piallat M-L, Walpoth BH, Möller M (2013). Eur J Pharm Biopharm.

[63] Wu W, Allen RA, Wang Y (2012). Nat Med.

[64] Lee K-W, Johnson NR, Gao J, Wang Y (2013). Biomaterials.

[65] Allen RA, Wu W, Yao M, Dutta D, Duan X, Bachman TN, Champion HC, Stolz DB, Robertson AM, Kim K, Isenberg JS (2014). Biomaterials.

[66] Gauvin R, Guillemette M, Galbraith T, Bourget J-M, Larouche D, Marcoux H, Aubé D, Hayward C, Auger FA, Germain L (2011). Tissue Eng, Part A.

[67] Wystrychowski W, Cierpka L, Zagalski K, Garrido S, Dusserre N, Radochonski S, Mcallister TN, L’heureux N (2011). J Vasc Access.

[68] Davies MG, Hagen P-O (1993). Ann Surg.

[69] Kakisis JD, Liapis CD, Breuer C, Sumpio BE (2005). J Vasc Surg.

[70] Wu KK, Thiagarajan P (1996). Annu Rev Med.

[71] Chan-Park MB, Shen JY, Cao Y, Xiong Y, Liu Y, Rayatpisheh S, Kang GC-W, Greisler HP (2009). J Biomed Mater Res – Part A.

[72] Korff T, Kimmina S, Martiny-Baron G, Augustin HG (2001). FASEB J.

[73] Tschoeke B, Flanagan TC, Cornelissen A, Koch S, Roehl A, Sriharwoko M, Sachweh JS, Gries T, Schmitz-Rode T, Jockenhoevel S (2008). Artif Organs.

[74] Niklason LE, Abbott W, Gao J, Klagges B, Hirschi KK, Ulubayram K, Conroy N, Jones R, Vasanawala A, Sanzgiri S, Langer R (2001). J Vasc Surg.

[75] Sorokin V, Vickneson K, Kofidis T, Woo CC, Lin XY, Foo R, Shanahan CM (2020). Front Immunol.

[76] Rickel AP, Deng X, Engebretson D, Hong Z (2021). Mater Sci Eng C.

[77] Niklason LE, Gao J, Abbott WM, Hirschi KK, Houser S, Marini R, Langer R (1999). Science (80).

[78] Quint C, Kondo Y, Manson RJ, Lawson JH, Dardik A, Niklason LE (2011). Proc Natl Acad Sci.

[79] Mallis P, Kostakis A, Stavropoulos-Giokas C, Michalopoulos E (2020). Bioengineering.

[80] Leal BBJ, Wakabayashi N, Oyama K, Kamiya H, Braghirolli DI, Pranke P (2021). Front Cardiovasc Med.

[81] Gui L, Niklason LE (2014). Curr Opin Chem Eng.

[82] Kristofik NJ, Qin L, Calabro NE, Dimitrievska S, Li G, Tellides G, Niklason LE, Kyriakides TR (2017). Biomaterials.

[83] Mackay CDA, Jadli AS, Fedak PWM, Patel VB (2022). Diagnostics.

[84] Campagnolo P, Cesselli D, Al Haj Zen A, Beltrami AP, Kra N, Katare R, Angelini G, Emanueli C, Madeddu P (2010).

[85] Tigges U, Komatsu M, Stallcup WB (2013). J Vasc Res.

[86] Zhang L, Issa Bhaloo S, Chen T, Zhou B, Xu Q (2018). Circ Res.

[87] Beamish JA, He P, Kottke-Marchant K, Marchant RE (2010). Tissue Eng – Part B Rev.

[88] Villalona GA, Udelsman B, Duncan DR, Mcgillicuddy E, Sawh-Martinez RF, Hibino N, Painter C, Mirensky T, Erickson B, Shinoka T, Breuer CK (2010). Tissue Eng Part B Rev.

[89] Huynh TN, Tranquillo RT (2010). Ann Biomed Eng.

[90] Syedain ZH, Meier LA, Lahti MT, Johnson SL, Tranquillo RT (2014). Tissue Eng, Part A.

[91] Roh JD, Brennan MP, Lopez-Soler RI, Fong PM, Goyal A, Dardik A, Breuer CK (2007). J Pediatr Surg.

[92] Godbey WT, Stacey Hindy BS, Sherman ME, Atala A (2004). Biomaterials.

[93] Hsu S-H, Tsai I-J, Lin D-J, Chen DC (2005). Med Eng Phys.

[94] Kilkenny C, Browne WJ, Cuthill IC, Emerson M, Altman DG (2010). PLoS Biol.

[95] Seliktar D, Black RA, Vito RP, Nerem RM (2000). Ann Biomed Eng.

[96] Nieponice A, Soletti L, Guan J, Deasy BM, Huard J, Wagner WR, Vorp DA (2008). Biomaterials.

[97] Laterreur V, Ruel J, Auger FA, Vallières K, Tremblay C, Lacroix D, Tondreau M, Bourget J-M, Germain L (2014). J Mech Behav Biomed Mater.

[98] Campagnolo P, Cesselli D, Al Haj Zen A, Beltrami AP, Kränkel N, Katare R, Angelini G, Emanueli C, Madeddu P (2010). Circulation.

[99] Armstrong JPK, Burke M, Carter BM, Davis SA, Perriman AW (2016). Adv Healthcare Mater.

[100] Zhang WH, Day GJ, Zampetakis I, Carrabba M, Zhang Z, Carter BM, Govan N, Jackson C, Chen M, Perriman AW (2021). ACS Appl Polym Mater.

[101] Fernández-Colino A, Wolf F, Rütten S, Schmitz-Rode T, Rodríguez-Cabello JC, Jockenhoevel S, Mela P (2019). Front Bioeng Biotechnol.

[102] Ghorbel MT, Jia H, Swim MM, Iacobazzi D, Albertario A, Zebele C, Holopherne-Doran D, Hollander A, Madeddu P, Caputo M (2019). Biomaterials.

