# Supplementary material for: Development of a Novel Hierarchically Biofabricated Blood Vessel Mimic Decorated with Three Vascular Cell Populations for the Reconstruction of Small-Diameter Arteries
Source: Adv Funct Mater. Author manuscript; Available in PMC 2024 Sep 10. (PMC7616429; doi:10.1002/adfm.202300621)
Supplement: Supporting Information [file EMS198235-supplement-Supporting_Information.pdf]

Supporting Information

Supporting Information is available from the Wiley Online Library or from the author.

© 2023 The Authors. Advanced Functional Materials published by Wiley-VCH GmbH

16163028, 2024, 7, Downloaded from https://onlinelibrary.wiley.com/doi/10.1002/adfm.202300621 by University Of Bristol Library, Wiley Online Library on [24/09/2024]. See the Terms and Conditions (https://onlinelibrary.wiley.com/terms-and-conditions) on Wiley Online Library for rules of use; OA articles are governed by the applicable Creative Commons License
